# University-Based Teleradiology in the United States

**DOI:** 10.3390/healthcare2020192

**Published:** 2014-04-15

**Authors:** Tim B. Hunter, Elizabeth A. Krupinski

**Affiliations:** 1 Department of Medical Imaging, College of Medicine, University of Arizona, Tucson, AZ 85724, USA; E-Mail: krupinski@radilogy.arizona.edu; 2 Arizona Telemedicine Program, College of Medicine, University of Arizona, Tucson, AZ 85724, USA

**Keywords:** telemedicine, teleradiology, university-based radiology, academic medicine

## Abstract

This article reviews the University of Arizona’s more than 15 years of experience with teleradiology and provides an overview of university-based teleradiology practice in the United States (U.S.). In the U.S., teleradiology is a major economic enterprise with many private for-profit companies offering national teleradiology services (*i.e.*, professional interpretation of radiologic studies of all types by American Board of Radiology certified radiologists). The initial thrust for teleradiology was for after-hours coverage of radiologic studies, but teleradiology has expanded its venue to include routine full-time or partial coverage for small hospitals, clinics, specialty medical practices, and urgent care centers. It also provides subspecialty radiologic coverage not available at smaller medical centers and clinics. Many U.S. university-based academic departments of radiology provide teleradiology services usually as an additional for-profit business to supplement departmental income. Since academic-based teleradiology providers have to compete in a very demanding marketplace, their success is not guaranteed. They must provide timely, high-quality professional services for a competitive price. Academic practices have the advantage of house officers and fellows who can help with the coverage, and they have excellent subspecialty expertise. The marketplace is constantly shifting, and university-based teleradiology practices have to be nimble and adjust to ever-changing situations.

## 1. Introduction

Teleradiology is the practice of radiology through the remote transmission and viewing of diagnostic radiology images comprising patient exams. The teleradiologists providing the interpretation of the transmitted studies are at a location separate from where the imaging studies were performed. Teleradiologists may provide a final written interpretation or they may provide a preliminary interpretation with the final interpretation provided later, typically by an in-house radiologist at the same location where the patient had the study. Teleradiologic studies cover the conventional sweep of radiologic procedures, including standard radiography (*i.e.*, X-rays), computed tomography (CT), ultrasound (US), magnetic resonance imaging (MRI), and nuclear medicine.

Teleradiology is big business in the United States (Table 1). There are several hundred private for-profit teleradiology companies that employ radiologists to provide teleradiologic interpretations. Surveys a few years ago showed nearly one-half of all radiology practices used or provided teleradiology services [1,2].

**Table 1:** Selected United States teleradiology vendors.

Cleveland Clinic eRadiology
Direct Radiology
Rays
StatRad
24/7 Radiology
Pediatric Radiology of America
NEXXRAD
VRad Alliance

Teleradiology in its earliest days 10–15 years ago traditionally meant coverage of emergent or urgent after-hours studies by a radiologist who was not only physically remote from the patient’s location but also was not financially associated with the radiologists providing routine in-house coverage. Since the driver for teleradiology was the coverage of after-hours studies (nights, weekends, holidays), those who provided such services came to be called “nighthawks” after a group of birds that feed on the wing in the early evening or pre-dawn hours. *Nighthawk* refers not only to teleradiologists working after-hours, but it also refers to those private for-profit corporations who employ teleradiologists.

At the current time, the definition of a nighthawk or teleradiologist is somewhat blurred. Many larger radiology practices provide their own after-hours coverage, sometimes with their group radiologists being in-house and sometimes with their group radiologists situated remotely. These individuals may be called “nighthawks” or teleradiologists. Also, some of the larger private for-profit teleradiology providers offer daytime services for routine or subspecialty coverage, and some of them even seek to replace traditional in-house practices, which has caused considerable consternation in the radiologic community [3,4,5,6,7,8,9,10,11]. In general, the term teleradiologist still usually means someone remote from where the patient’s imaging was performed and someone not otherwise financially connected to the in-house radiology group or the medical center itself.

## 2. University-Based (Academic) Radiology

### 2.1. Medical Reimbursement for the United States

The United States does not have a single payer system for healthcare found in many developed countries, such as those in Western Europe. However, significant healthcare monies in the United States are spent by the Federal and state governments for veterans benefits, Medicare (for all citizens 65 and older) and for Medicaid for low income adults and children. Most working adults are provided private health insurance as a benefit from their employer. There is also a very small percent of self paying patients who pay for their healthcare directly not going through a government or insurance third party.

Self pay covers two extremes, those who are quite wealthy and can easily afford to pay “cash on the barrelhead” for any sort of medical treatment or hospitalization. On the other extreme are those who do not have health insurance and struggle to pay any medical bills. The term “self pay” is often used negatively to mean no payment for services rendered.

The relative mix in a given academic medical center of Medicare, Medicaid, private insurance, and self pay patients varies greatly. This payer mix often determines the financial health of a medical center or medical practice. In general, private insurance is the preferred payer as reimbursement is often higher than that provided by government related programs. For some specialties and office practices, Medicare may not pay one’s cost to provide service. Some medical centers and practices will not take Medicare, Medicaid, or self pay patients, but that is unusual for academic medical centers which usually take all comers.

In most academic medical centers each department is responsible for its own financial welfare, particularly with regard to physician reimbursement. Physician reimbursement varies between specialties and is not evenly distributed across the medical practice. Each university academic practice is different in this regard, and no broad generalizations can easily be made.

Unfortunately, not only is the chair of an academic radiology department competing for business with other radiology practices in town or across the country in the case of teleradiology, but he or she may be competing for institutional resources with other departments in the medical center and has to be forever on guard to protect departmental finances.

The payer mix changes from year to year and is considerably different from one academic medical center to another. The approximate payer mix for our institution is Medicare 38%, Medicaid 17%, commercial private health insurance 31%, self pay 11%, and Tricare (veterans and military) 3%.

### 2.2. Academic Medicine

In the United States the terms *academic radiology* and *university-based radiology* are typically used interchangeably. Another term frequently encountered is *teaching hospital*. A teaching hospital is often, but not always, affiliated with a university. A teaching hospital has one or more formal programs for hospital-based teaching of medical students, students in allied health professions, or house officers in nationally accredited medical specialty training programs. The Association of American Medical Colleges (AAMC) represents 141 accredited United States and 17 Canadian medical schools that award the MD degree. Under the AAMC umbrella there are also 400 major teaching hospitals and health systems, including 51 Department of Veterans Affairs medical centers and approximately 90 academic and scientific societies [12]. There are also 29 colleges of osteopathic medicine that award the Doctor of Osteopathic Medicine (DO) degree, 23 of which are private and six public [13].

Diagnostic radiology post graduate year 1 (PGY-1) is a transitional year after medical school graduation. It is sometimes thought of as an “internship,” but that term is not officially used. Very often PGY-1 is in a different location than the diagnostic radiology residency itself which consists of 4 years (PGY-2 through PGY-5). In 2013 there were 158 diagnostic radiology residencies offering 979 positions in a national matching system [14]. All of these programs are based in teaching hospitals, the majority of which are either owned or affiliated with a university having a college of medicine. Some radiology residency programs are free-standing or situated in a large teaching hospital in a private or public institution that does not have a university affiliation. For the purpose of this article, an academic or university-based radiology residency is defined as a residency program in a teaching hospital(s) owned or closely affiliated with a private or public university having a college of medicine. Such a department of radiology is defined as an academic or university based department.

### 2.3. Academic Mission; Academic Economics

The mission of an academic department of radiology is three fold: to provide patients with outstanding evidence-based radiologic practice; to provide education to medical students, radiology residents, other house officers, and associated medical personnel; and to further medical knowledge through basic science and clinical research.

Since university-based radiologists have important teaching and academic missions, they cannot devote as much time to clinical practice and thus receive lower salaries than those in private practice. Ideally, the mission of an academic radiology department is not focused as much on the business aspects of radiologic practice as those in private practice. Unfortunately, the reality of medical practice in the United States at most academic institutions is that academic radiologists and academic physicians in general must earn their keep through clinic practice with the remuneration for teaching and academic effort being minimal compared with the remuneration for clinical work [15].

The chair of a university based radiology department spends most of his or her waking time trying to keep the department solvent. SCARD (Society of Chairs of Academic Radiology Departments) advances the broad mission of academic radiology, but much of its effort is directed towards helping its members keep their departments financially sound [16]. Funds that flow towards academic departments of radiology for teaching and research are usually diminutive compared to clinical dollars generated by the department. While academic radiologists do not command the salaries found in private practice, and while the job situation for radiologists in the United States has become very challenging, radiologists nevertheless are expensive with high salaries compared to most medical specialties.

### 2.4. Why Academic University Based Teleradiology?

University-based radiology practices compete in the marketplace with everyone else, including teleradiology. A number of academic radiology departments have a teleradiology practice (Table 2). A department chair must attract and keep talented faculty, especially junior faculty who often leave within a few years of being hired for the perceived greener pastures of private practice. There is a common perception in academic radiology that the clinical workload is similar to private practice, but the salaries are less, and there is less free time due to the teaching and academic (publication and research) demands placed on faculty [15]. Despite this, however, many radiologists prefer the advantages and demands of working in the academic environment.

**Table 2:** Selected University Based Teleradiology Practices.

Academic Institution	Reference
Cleveland Clinic	[17]
Emory University	[18]
Massachusetts General Hospital	[19]
UC Davis Health System	[20]
University of Arizona	[21]
University of Pittsburgh	[22]
University Radiology Subspecialty Imaging in New Jersey	[23]
UC San Diego	[24]
University of South Alabama College of Medicine	[25]
University of Virginia School of Medicine	[26]
Veterans Administration Palo Alto Healthcare System	[27]

Department chairs continually look for new sources of clinical revenue, particularly those that have low associated costs and those that fit the academic mission of the department. Teleradiology offers many potential advantages to an academic radiology department. There are innumerable small hospitals, medical centers, and clinics that desire increased radiologic coverage, especially for after-hours and for subspecialty interpretations [28]. In many academic radiology departments there is not enough clinical work to completely occupy the available faculty, and paring down the faculty size is undesirable not only from the human point of view but because of the harm it might cause to the teaching and academic missions of the department.

Academic departments already have residents/fellows on call after-hours and faculty are often also present at night (at least in the early evening). Thus one successful model for teleradiology uses senior residents/fellows during these after hour periods to cover the teleradiology service, calling upon the faculty for difficult case interpretations as needed. The advantage is that the extra teleradiology cases provide enough work to fill the time of the residents/fellows, and it also provides additional cases that are quite valuable from an educational perspective as they often come from very different patient populations than those seen at the academic institution. The possible drawback of this model is that not all referring sites are willing to accept reports made by residents and only will contract if faculty interpret the cases.

One very costly and complex aspect of all radiology practices is billing and collections. These costs can be quite onerous in an academic practice. The billing fee rate as a percent of net clinical revenue ranges from 2.5% to greater than 10% averaging around 7% according to one of the academic surveys listed on the SCARD website. Corporate overhead ranged from 0%–20% with most institutions being in the 5%–10% range [16]. An earlier survey showed the combination of Dean’s tax (*i.e.*, typically the Dean of the College of Medicine takes a percentage of the clinical revenue to support other academic activities) and billing overhead ranged from 9%–30%.

Any reduction in the billing costs and associated academic taxes, such as the Dean’s tax or the university tax, greatly increase a department’s net revenue. It might also increase revenue as fewer personnel are required for billing and collections. Simplified billing and collections is one of the strong selling points for an academic department of radiology to start a teleradiology practice, because the most frequent payment scheme for a teleradiology provider is to be paid a set amount per case read [28]. This is paid directly to the teleradiology vendor by a contracting hospital or clinic. The per-case charge varies depending on the nature of the study, for example simple radiograph *versus* complex MRI study. It often also depends on whether it is an urgent or a routine study. In most cases, the teleradiology vendor provides an invoice detailing the numbers and types of cases read in a given period along with a charge sheet and a summary of the total charges, while the contracting facility confirms this information and pays the invoice [28].

The teleradiology provider does not bill patients and does not charge differently for patients with Medicare, Medicaid, self-payment, and private insurance. Not only are billing costs and other associated taxes often reduced or eliminated for an academic radiology department, the department is paid for all cases read. Just a small percent change in the collection rate for cases read can mean a large net income swing for an academic radiology department.

A variation on this teleradiology payment scheme is for a teleradiology provider to read all designated studies from a hospital or clinic for a set fee for a given period, typically an annual charge for radiologic services. There may be provisions for increased payment if the workload exceeds a given number of studies, and there may be bonus and penalty provisions based on quality metrics, such as report turnaround times [28]. Contract negotiations usually revolve around the teleradiology provider’s case charges for interpretations with the starting point being the Medicare radiology professional fee payment schedule. The teleradiology provider would like to receive 125% or more of the Medicare fee schedule, while the contracting entity may want to pay only 75% or less.

## 3. The University of Arizona Teleradiology Experience

### 3.1. Arizona Telemedicine Program

In 1996 the Arizona State Legislature budgeted $1.2 million to establish and fund the first year of operation of the Arizona Rural Telemedicine Network (now called the Arizona Telemedicine Network) with the hub of the network at the Arizona Health Sciences Center (AHSC) in Tucson, Arizona. AHSC is an integral part of the University of Arizona and is Arizona’s only academic health sciences center. The Arizona Rural Telemedicine Network operation was broadened to become the Arizona Telemedicine Program “to oversee and coordinate telemedicine clinical, education, research and telecommunications programs, as well as to operate the Arizona Telemedicine Network” [29]. The Arizona Telemedicine Program provides an extensive range of clinical services and teaching for the citizens of Arizona, and it conducts extensive research in the fields of telemedicine and telehealth. The University of Arizona considers the Arizona Telemedicine Program to be a very important part of the University’s mission to the citizens of Arizona.

### 3.2. University of Arizona Teleradiology

The University of Arizona teleradiology program was established in 1997 because of the radiology department’s leadership’s close association with the leadership of the Arizona Telemedicine Program. The radiology department had been and continues to be a world leader in digital imaging, and its research collaborations with departments like Electrical and Computer Engineering laid the early foundations for the creation of the Arizona Telemedicine Network. By 1997, the technology for digital image transmission and reception had matured enough to permit the practical establishment of teleradiology throughout the United States, and early pilot studies at the University of Arizona demonstrated that teleradiology was not only feasible but highly practical and useful [30,31]. The initial impetus for teleradiology was for after-hours coverage and for provision of radiologic services to small remote, rural hospitals and clinics with limited access to radiologists [28,32].

At that time the department was charged an 11% billing fee and various university assessments added another 8% tax onto net revenues. The departmental leadership was able to structure an agreement for teleradiology revenue with the University Physicians, Incorporated (now University Physicians Healthcare), the clinical practice plan for the physicians at the College of Medicine at the University of Arizona. This agreement allowed the radiology department to start a teleradiology program with direct billing to medical clinics and hospitals for which it provided teleradiology services. The department simply billed for the cases read on a per case basis as outlined above. There were no billing charges per se, and the associated university assessments were somewhat reduced such that the teleradiology service became one of the most profitable clinical operations in the department, with a billing cost of approximately1% of net revenue.

From 1997 to 2009 the teleradiology practice of the department greatly expanded. More than 1,000,000 teleradiology cases have been officially interpreted, and more than 30 sites have been covered at various times during the years. Most teleradiology practices, including university-based practices, experience considerable turnover in clientele from year to year. New sites are added periodically, and older clients are lost or dropped from time to time for several reasons. Sometimes, clients increase their in-house radiologist coverage and no longer need teleradiology services. At other times, they become dissatisfied with their current teleradiology coverage and contract with a new vendor. This dissatisfaction may be a combination of too expensive pricing, long turnaround times for reports, and/or perceived poor quality of the radiology coverage. There are also instances of changes in the leadership at local hospitals where the new leadership wishes to give contracts for teleradiology, emergency medicine, and other services to favored providers having previous associations with the leadership.

In our academic teleradiology practice we concentrated on locations in Arizona and New Mexico to further the mission of the University of Arizona for outreach to the citizens of Arizona, and because we were most familiar with the medical entities in our own state and the neighboring state of New Mexico. Some of our clients have medical facilities in both states and our teleradiologists are licensed in both states and credentialed at each of the referring institutions. Our teleradiology program was established both to provide professional income to the department and radiologic services to communities without dedicated full time on-site radiologists [28]. The bulk of our teleradiology practice has been in federal facilities—Indian Health Service hospitals, Public Health Service hospitals, and medical clinics associated with Native American tribes. We also serve a county penal institution, a private urology practice, and a private MRI imaging center. At one time we provided teleradiology and daytime coverage to a local proprietary heart hospital, but we did not renew that contract on its expiration as we felt it produced too great a daytime and evening burden on the residents and attending staff.

Our teleradiology practice since its inception has provided a combination of preliminary and final readings for all imaging modalities, including nuclear medicine and fluoroscopic studies. Until 2012, we provided a monthly two-day rotation of selected radiology faculty who traveled to two of our customers’ facilities to provide on-site fluoroscopy coverage. Almost all readings performed during routine working hours are by attending faculty radiologists who provide a final interpretation. The majority of the after-hours interpretations are by senior diagnostic radiology residents who give a preliminary reading which is followed later by a final reading by an attending faculty radiologist [28].

The Department of Radiology (now Department of Medical Imaging) residents who provide the after-hours preliminary readings are senior residents who volunteer for this after-hours “moonlighting” which pays a competitive compensation. These residents are credentialed with our academic practice plan and have unrestricted medical licenses in the State of Arizona. There is an internal departmental peer review program for both the resident and the faculty interpretations [33]. There is a system in place for rapid identification of significant resident after-hours interpretation errors and for rapid written and phone notification of the physician or responsible patient healthcare provider where an erroneous interpretation either by a resident or faculty member might cause patient harm. Internal departmental statistics for peer review for both residents and faculty are constantly monitored with significant errors under 1% that are consistent with those found in other institutions [34,35].

At one time, teleradiology was approximately 30% of the department’s net revenue. It has decreased to fewer than 10% at the present time as new departmental leadership has concentrated on increasing the department’s clinical and basic science research footprint and increasing departmental revenues through growth of the in-house practice. The present leadership is still supportive of having a teleradiology practice (now called outreach imaging) but wants to be more selective in obtaining contracts which have a higher percent of cross-sectional imaging (CT, MRI) and are somewhat more remunerative.

## 4. Building a Successful University Based Teleradiology Practice

### 4.1. Advantages

The prime reason for a university radiology department to establish a teleradiology practice is to increase its income. The more studies read the greater the net revenue. As discussed previously, the billing costs and other university practice costs tend to be significantly less for teleradiology than for the in-house side of the practice. Depending on the teleradiology clients, there may be a significant advantage for teaching and research missions as the teleradiology case mix may be completely different than everyday practice. Our teleradiology practice reflects the large incidence of diabetes and its complications found in Native American communities. There are many studies showing instances of osteomyelitis, Charcot arthropathy, pneumonitis, and atherosclerotic disease. The congenital and developmental diseases and degenerative diseases tend to be more complex and different in Native American and rural communities than in the typical academic practice.

While our in-house university practice is in a medical center with a Level 1 trauma center, we have seen many unusual traumatic injuries as part of our teleradiology practice. The Native American communities and rural community hospitals we serve do not have a high level of everyday trauma, but they do have unusual traumatic injuries related to horse, cattle, and sheep ranching, rodeo events, and the occasional severe automobile accident on a remote stretch of the interstate highway system.

Since many of our teleradiology studies are initially interpreted by a senior resident, they broaden the residents’ learning experiences, and during the day, the teleradiology cases are incorporated into the academic practice in each section of the department. This is an excellent learning experience for our residents and fellows. They learn how to read studies from remote locations with all the associated challenges of connectivity, report generation, and obtaining proper history as well as making sure a critical finding is directly communicated to the patient’s providers in a timely fashion.

Teleradiology reports have to be brief and to the point [36]. Most of our client locations have CT imaging and standard radiography, but many do not have ultrasound or MRI imaging. Some do not even have CT imaging, and most do not have nuclear medicine imaging. Long rambling reports with no firm conclusions and ill thought out recommendations for further imaging are of no use. Thus, diagnosing pneumonitis in an infant, appendicitis in an adolescent or acute cholecystitis in an elderly adult, for example, without clear radiographic findings burdens the patient and his or her physician with an unnecessary, possibly harmful treatment plan of action. A patient may be transported over 100 miles in the middle of the night to a larger medical center for more advanced treatment based on our readings.

Residents and inexperienced faculty in particular may succumb to the temptation to overcall marginal radiologic findings to cover themselves from missing a diagnosis. Some cases are quite difficult, and it is incumbent on the interpreting radiologist on the spot to contact the treating physician to discuss the patient’s findings [28]. It may necessitate consultation with a colleague or attending radiologist, and sometimes one has to admit the findings are equivocal and difficult to interpret. Recommending further imaging is frequently the case in daily radiologic practice. It should be based on sound radiologic and clinical findings. In a teleradiology practice recommending an MRI study or a nuclear medicine exam for a somewhat frivolous reason may have major costs and inconveniences for the patient and medical facility far greater than in an urban area.

Residents and faculty have to be cautious when interpreting ultrasound examinations for which they are not physically present and not able to view or participate in the real-time portion of the examination. However, one cannot simply refuse to interpret freeze frame images from remotely obtained ultrasound examinations as a matter of principle, because this will endanger the contract for that facility as the facility will quickly look for another provider. More importantly, it will deprive patients of imaging expertise. The majority of ultrasound examinations transmitted is interpretable and provides helpful information for patient treatment. Gallstones, abscess formation, aneurysms, large hematomas, pleural effusions, ascites, liver masses, and so forth are readily apparent. One becomes adept at being cautious when the images are not of high quality and when one is uncertain as to the actual scanning technique.

Our teleradiology practice contributes significantly to the teaching mission of the department in addition to its economic benefit. It also contributes to the academic mission of the department as well. Residents, fellows, and faculty see a case mix quite different from the usual academic practice. They learn how to interpret studies from remote locations with its challenges and its satisfaction of helping underserved populations. Departmental faculty are major investigators examining telemedicine and teleradiology both from a technical point of view and from a patient service point of view ([30,31,37,38,39,40]). Moreover, it is likely most of our residents and fellows will join a practice in which they will interpret studies from remote locations.

### 4.2. Disadvantages and Challenges

Teleradiology practice has considerable challenges for a university-based radiology department. The monies generated may not be worth the inconvenience engendered by the increased workload which is often significant after-hours. This may distort the departmental work schedule and put additional stress on residents, fellows, and faculty who typically handle the after-hours cases. Academic practices pride themselves on their subspecialty expertise, but it is difficult to provide subspecialty expertise around the clock. Our increased teleradiology workload was initially put on the backs of the on-call residents, the nighttime faculty, and the faculty covering the morning work slots. The morning faculty received a large number of preliminary readings from the prior evening not only having to provide final reports but also having to correct any erroneous readings from on-call residents and having to contact remote physicians to give them amended reports for their patients.

Our early teleradiology contracts were signed by the departmental leadership with little or no consultation from the affected faculty. As is typical with many academic practices, the infrastructure was somewhat lacking. Initially, there were no additional resources given to the residents and faculty reading studies from the teleradiology sites. In fact, the teleradiology workstations were in inconvenient locations, the software was clumsy, and faculty had to print out their reports and individually fax them to the teleradiology sites!

Any teleradiology practice typically covers multiple institutions concurrently on a given work shift. Cases can sometimes backup, and our overworked residents and faculty often vented their frustrations on a physician inquiring why it was taking so long to receive a patient report. It does not engender good will to tell a hard working physician in a remote location with a very sick patient that his patient’s study will be read in the next hour or so after cases from other locations are cleared first. All of these challenges were gradually overcome by hiring more faculty, by separating the on-call in-house workload from the after-hours teleradiology work, and by constructing a voluntary paid teleradiology moonlighting practice for our senior residents as noted above. A daytime teleradiology coordinator was hired to facilitate the teleradiology practice both for the in-house teleradiologists and for the teleradiology sites.

As our teleradiology practice evolved, more user friendly software was installed, and several workstations were added making it considerably more convenient to report teleradiology cases. The faculty participated fully in the addition of new departmental contracts, teleradiology and otherwise, and some sites were dropped from our teleradiology practice due to financial or coverage considerations. The finances and work schedule for the department were openly discussed at faculty meetings, and the faculty had considerable say in departmental operations creating more of a buy-in and vested interest in expanding our teleradiology practice.

In our practice most of the after-hours coverage is from different radiologists from night to night. This is generally not a problem, but it can lead to an inconsistency in readings [41]. This has been overcome in part by having the same faculty on during the regular work day and by having each section covering its portion of the teleradiology practice. This daytime consistency has proved very useful as physicians from the teleradiology locations are used to calling during the day to consult with our faculty. We also found that our moonlighting teleradiology residents became quite familiar with our teleradiology sites and became adept at conveying important patient information to the patients’ physicians. The residents and teleradiology faculty take the time to review previous patient studies and reports.

### 4.3. Patient Reports

There should be an official written report whether preliminary or final for every patient teleradiology study. A lost report with critical patient findings is one of the radiologist’s worst nightmares. Every teleradiology practice has to ascertain how their reports actually arrive at the originating site and become available to the treating physician. This is far from a trivial consideration. Any successful teleradiology practice has to be prepared to pay for special software so its reports can be written to the electronic records at the originating sites. University based practices sometimes seem less willing to invest in such infrastructure than private teleradiology vendors, looking at their teleradiology practice as a side venture that should not require too much time, effort, or money. This sort of attitude will eventually doom the practice. It is best to never assume the radiology report will reach the proper place unless there is an active process for establishing report flow dynamics. There should also be a formal process for the prompt reporting of critical findings (Table 3), such as a tension pneumothorax, a ruptured aneurysm, free intra-abdominal gas, and an unstable spine fracture, with oral physician to physician communication in most instances.

**Table 3:** Critical Radiologic Findings ([28]).

1. Large pneumothorax; tension pneumothorax
2. Unexpected or very large pulmonary embolus
3. Large or unexpected intracranial hemorrhage
4. Large pericardial effusion
5. Dissection of the aorta
6. Unstable spine fracture
7. Free intra-abdominal gas
8. Unexpected fetal anomaly
9. Unexpected lung mass

### 4.4. Putting It Together for a Successful University Based Teleradiology Practice

In the United States and elsewhere it is a tough competitive world, and a university-based teleradiology practice is by no means guaranteed to be successful. If it is imposed on the faculty and if there are insufficient resources both technical and personnel, it will fail. It cannot be imposed on the faculty from above, and faculty cannot be forced to travel to remote sites unwillingly. The faculty should not perceive the teleradiology as added extra duty with no reward. A teleradiology practice is not for every academic radiology department. However, under the proper circumstances, it can be financially rewarding and contribute significantly to the department’s service, teaching, and research missions. Table 4 lists general recommendations for a successful university based teleradiology practice.

**Table 4:** Recommendations for a Successful University-Based Teleradiology Practice.

Subspecialty based practice with consistent faculty and resident participation on a daily basis
Concise, well written reports with definitive conclusions and recommendations where appropriate
Round the clock support services-teleradiology technical help line; physician’s teleradiology resource line
Consistent delivery of patient reports to the originating site with formal process for the prompt communication of critical findings
Proper faculty licensing, credentialing, and peer review
Peer review results available to contracting sites obeying confidentiality and peer review protection
Easy, formal process for resolving complaints concerning erroneous reports or other problems
Competitive pricing
Regular communication and periodic on-site visits

Teleradiology should be an intimate part of a university based radiology practice rather than being an added on side business. One has to provide good readings with short reports to the point, ideally with the impression first in the report. There must be rapid turnaround of cases, and there has to be excellent urgent and emergency coverage. Good daily interactions with originating site providers is a requisite as is a technical help line for the originating sites when there are problems with the transmission of images or the receipt of reports. There should also be a physician’s resource line or a call center for physicians wishing to speak with a teleradiologist or inquire about patient studies. It is also beneficial for at least one representative from the department to visit the referring sites on a regular basis to discuss potential issues and challenges or simply to reaffirm commitments and “put a face” on the teleradiology practice.

It goes without saying that any university-based teleradiology practice has to have its faculty credentialed at each facility for which studies are interpreted. The faculty should have proper medical licensure and be covered by adequate malpractice insurance. There should be a formal peer review process for the continued evaluation of the faculty, residents, and fellows. The results of this peer review process should be available to the facilities contracting teleradiology services in a manner consistent with legal peer review protection and confidentiality according to applicable state laws. There should also be a formal mechanism to resolve complaints or queries concerning a specific radiologic report or possible error by an interpreting radiologist.

## 5. Conclusions

Many U.S. university-based academic departments of radiology provide teleradiology services either as part of their academic mission or, more commonly, as an additional for profit business to supplement departmental income. Since academic based teleradiology providers have to compete in a very demanding marketplace, their success is not guaranteed. They must provide timely high quality professional services for a competitive price. Academic practices have the advantage of house officers and fellows who can help with the coverage, and they have excellent subspecialty expertise. Many academic practices also have sophisticated credentialing and peer review programs to ensure a high quality practice. The marketplace is constantly shifting, and university based teleradiology practices have to be nimble and adjust to ever changing situations.
